# A Review on Finerenone and Heart Failure with Preserved Ejection Fraction (HFpEF)

**DOI:** 10.2174/011871529X457971260121071610

**Published:** 2026-03-18

**Authors:** Sidhartha Gautam Senapati, Debabrata Mukherjee

**Affiliations:** 1 Department of Internal Medicine, Texas Tech University Health Sciences Center, El Paso, TX, USA;; 2 Division of Cardiology, Department of Internal Medicine, Texas Tech University Health Sciences Center, El Paso, TX, USA

**Keywords:** Heart failure with preserved ejection fraction, HFpEF, finerenone, mineralocorticoid receptor antagonist, nonsteroidal MRA, FINEARTS-HF, chronic kidney disease, SGLT2 inhibitor, NT-pro BNP, cardiorenal protection, hyperkalemia, cardiovascular outcome

## Abstract

**Introduction:**

Heart failure with preserved ejection fraction (HFpEF) accounts for about half of all heart failure cases and is difficult to treat because it has many causes and limited treatment options. Finerenone is a newer nonsteroidal mineralocorticoid receptor antagonist (MRA) that is more selective, distributes evenly between the heart and kidneys, and leads to fewer hormone-related side effects than older steroidal MRAs.

**Methods:**

This review examines results from key clinical trials, such as FIDELIO-DKD, FIGARO-DKD, and FINEARTS-HF, as well as recent studies that have followed patients and explored how finerenone works in HFpEF and heart failure with mildly reduced ejection fraction (HFmrEF). Studies published between 2020 and 2025 were found using PubMed, Embase, Scopus, and the Cochrane Library.

**Results:**

Clinical trials found that finerenone reduced the risk of cardiovascular events, including heart failure hospitalizations and cardiovascular death, regardless of ejection fraction. Biomarker analysis showed lower NT-pro BNP levels, improved diastolic function, and less cardiac remodeling. Finerenone also had strong anti-inflammatory and antifibrotic effects, maintained renal safety, and caused less hyperkalemia than steroidal mineralocorticoid receptor antagonists (MRAs). These benefits appeared in all groups, no matter their age, sex, kidney function, or use of sodium-glucose cotransporter 2 (SGLT2) inhibitors.

**Discussion:**

Finerenone provides cardiorenal protection by targeting key inflammatory and fibrotic pathways in HFpEF. Its favorable safety profile and additive effects with SGLT2 inhibitors support its integration into evolving guideline-directed therapy.

**Conclusion:**

Finerenone is a significant step forward in treating HFpEF, as it provides both heart and kidney protection. Current studies will help identify which patients benefit most and how it affects long-term results in more people with HFpEF.

## 1 INTRODUCTION

### 1.1 Overview of Heart Failure and HFpEF

Heart failure (HF) happens when the heart cannot pump enough blood to meet the body’s needs, even if filling pressures are normal or high. People with HF often feel short of breath, tired, and may have fluid buildup. These symptoms lead to high rates of illness and death around the world. One type, called heart failure with preserved ejection fraction (HFpEF), is diagnosed when someone has heart failure symptoms and a left ventricular ejection fraction (LVEF) of 50% or more [1]. HFpEF is getting more attention because it is becoming more common and is often hard to treat due to its complexity.

Heart failure with preserved ejection fraction differs from heart failure with reduced ejection fraction (HFrEF) in its development and response to treatment. HFrEF is mostly due to the problems with the heart’s pumping, changes in its shape, and higher activity of certain hormones. Treatments for HFrEF have been proven to reduce death rates. In contrast, HFpEF is mainly due to the heart having trouble relaxing, increased stiffness, inflammation in small blood vessels, and scarring of heart tissue. It often affects older adults who have other health conditions. Because of these differences, standard treatments for HFrEF do not work as well in HFpEF, underscoring the need for therapies that target inflammation, scarring, and the link between the heart and kidneys.

HFpEF makes up more than half of heart failure cases worldwide, showing its growing impact on older adults and people with other heart and metabolic conditions. Chronic kidney disease (CKD) and type 2 diabetes (T2DM) often occur alongside HFpEF, making the disease harder to manage. The relationship between HFpEF and CKD goes both ways and leads to worse outcomes, so treatment needs to be coordinated [2]. As the population ages and conditions like hypertension, obesity, and metabolic syndrome become more common, HFpEF is becoming a major public health issue with serious clinical and economic effects.

HFpEF is a complicated condition with several causes. It includes diastolic dysfunction, trouble with heart muscle relaxation, stiffer ventricles, microvascular inflammation, and fibrosis. Systemic endothelial dysfunction, ongoing mild inflammation, and neurohormonal activation also contribute to HFpEF development [3]. Unlike heart failure with reduced ejection fraction (HFrEF), which mostly affects how well the heart contracts, HFpEF is marked by changes like fibrosis and thickening of the heart muscle. These changes are often linked to high blood pressure and diabetes, which trigger pathways that cause fibrosis and inflammation.

### 1.2 Introduction to Mineralocorticoid Receptor Antagonists (MRAs)

The mineralocorticoid receptor (MR) is a nuclear hormone receptor that helps control electrolyte and fluid balance through aldosterone signaling, mainly in the kidney. In addition to these main roles, MR signaling is linked to the development of cardiovascular disease because it affects the heart, blood vessels, and kidneys. When MR is overactive, it can cause vascular inflammation, cardiac fibrosis, hypertrophy, and remodeling. These changes are key factors in heart failure. This knowledge supports the use of mineralocorticoid receptor antagonists (MRAs) as a treatment[4].

Historically, steroidal MRAs such as spironolactone and eplerenone demonstrated eﬀicacy in reducing mortality and hospitalizations in HFrEF patients. However, their broader use, particularly in HFpEF, is constrained by adverse effects, including hyperkalemia and steroid-related endocrinological side effects like gynecomastia and menstrual irregularities. These limitations have tempered enthusiasm for steroidal MRAs in the HFpEF population and highlighted the need for more selective agents [1].

This clinical gap motivated the development of nonsteroidal MRAs, among which finerenone has emerged as a novel therapeutic agent. Finerenone is distinguished by higher selectivity and affinity for MR, balanced cardiac and renal tissue distribution, and a favorable side-effect profile compared to the steroidal MRAs. Its pharmacological profile allows potent receptor blockage while mitigating off-target hormonal effects, rendering it promising in HFpEF, particularly in those with coexisting CKD and T2DM[5].

Steroidal mineralocorticoid receptor antagonists like spironolactone are effective for HFrEF, but it is uncertain how well they work for HFpEF. Side effects such as high potassium, worsening kidney function, and hormonal changes like gynecomastia and menstrual problems often limit their use. These concerns are especially relevant for HFpEF patients, who are usually older, often have chronic kidney disease, and typically take several medications. Steroidal MRAs can also accumulate in the kidneys, which may increase the risk of electrolyte problems without offering lasting antifibrotic benefits for the heart.

### 1.3 Rationale for Reviewing Finerenone in HFpEF

Although HFpEF is common and has a poor outlook, there have been fewer treatment advances compared to HFrEF, leaving many clinical needs unmet. The varied causes of HFpEF and challenges in running large trials have made it hard to find effective treatments [3]. Because finerenone has strong anti-inflammatory and antifibrotic effects that match the disease’s underlying mechanisms, it may be a promising option to fill this treatment gap.

Finerenone’s superior safety and tolerability, especially its lower rates of hyperkalemia and endocrine side effects, may allow for broader and longer use in older patients with HFpEF and other comorbidities[1]. The FIDELIO-DKD and FIGARO-DKD trials mainly focused on kidney outcomes in people with type 2 diabetes and chronic kidney disease. In these studies, cardiovascular outcomes like heart failure hospitalization were secondary, not primary, endpoints for heart failure [6]. These findings support the need for a thorough review of finerenone’s role in managing HFpEF.

## 2 METHODOLOGY

### 2.1 Search Strategy

We searched PubMed, Embase, Scopus, and the Cochrane Library for articles published between January 2020 and October 2025. Our search used keywords such as “finerenone”, “heart failure with preserved ejection fraction”, “HFpEF”, “nonsteroidal mineralocorticoid receptor antagonist”, and “FINEARTS-HF”, combined with AND and OR. We also reviewed the reference lists of selected papers and relevant reviews to find more studies. We included only peer-reviewed studies from the last five years (2020-2025), except for landmark trials or important mechanistic references.

### 2.2 Study Selection

Two reviewers separately checked titles and summaries to identify studies that met the criteria. We included full English articles that looked at the effectiveness, safety, or use of finerenone in HFpEF or HFmrEF. Eligible studies were randomized controlled trials, meta-analyses, or strong observational studies. We excluded studies with children, people without heart conditions, reviews without new clinical data, and reports that were not peer-reviewed or were duplicates. If the reviewers disagreed, they talked it over until they agreed.

### 2.3 Data Extraction and Synthesis

We gathered data from the studies using a standard form that noted how each study was designed, the number of participants, patient information, treatments given, and results such as deaths from heart problems, hospital stays for heart failure, lower NT-proBNP levels, and any side effects. We gave more weight to studies where people were randomly assigned to groups than to those that only observed participants. We combined information about how the treatments work and their real-life effects, comparing results from studies like FIDELIO-DKD, FIGARO-DKD, and FINEARTS-HF. We organized the evidence by how the treatments work, their outcomes, and their safety.

This structured and reproducible approach ensured comprehensive coverage of the available literature, minimizing bias and improving the robustness of the conclusions.

## 3 PHARMACOLOGICAL PROFILE OF FINERENONE

### 3.1 Molecular Mechanism and Selectivity

Finerenone is a third-generation, nonsteroidal mineralocorticoid receptor antagonist with unique pharmacodynamic properties that support its clinical use. It binds strongly and selectively to the mineralocorticoid receptor, which increases its effectiveness and reduces unwanted effects seen with steroidal MRAs like spironolactone and eplerenone[7]. This selectivity allows for more precise blocking of the receptor, helping to stop aldosterone-driven inflammation and fibrosis, which are important factors in cardiorenal remodeling.

Finerenone has a larger and unique chemical structure compared to spironolactone and eplerenone, which gives it different receptor-binding properties. As a result, it affects tissues in specific ways and changes downstream signaling, leading to strong anti-inflammatory and antifibrotic effects that help protect the heart [1]. Studies using network pharmacology and molecular docking have also shown that finerenone can interact with several key targets involved in heart and kidney disease, such as CASP3, MMP9, and EGFR. This supports its broad and varied way of working[8].

Finerenone is different from steroidal MRAs because it has a nonsteroidal structure, greater selectivity for mineralocorticoid receptors, and a balanced distribution between heart and kidney tissues. Unlike spironolactone and eplerenone, finerenone does not activate androgen or progesterone receptors, so it causes fewer endocrine side effects. Its balanced tissue penetration helps block harmful heart inflammation and fibrosis more effectively, while also reducing too much mineralocorticoid receptor blockade in the kidneys. This likely leads to fewer cases of hyperkalemia and better kidney tolerability.

### 3.2 Pharmacokinetics and Tissue Distribution

Finerenone is absorbed quickly and has high oral bioavailability, which leads to predictable levels in the body and allows for once-daily dosing [7]. The drug is mainly broken down in the liver and does not build up in the body, making it suitable for patients with different levels of kidney function.

Finerenone is unique because it spreads evenly between the heart and kidneys [5]. Steroidal MRAs, on the other hand, often collect more in the kidneys, which can lead to safety concerns and problems with electrolytes. Since finerenone is distributed more evenly, it helps protect both the heart and kidneys and lowers the risk of high potassium and kidney problems. As a result, finerenone generally has a better safety profile and causes fewer side effects than traditional MRAs[9].

### 3.3 Clinical Implications of Pharmacology

Finerenone has unique pharmacological features that make it effective for treating patients with HFpEF, CKD, and T2DM, as shown in several clinical trials. Its selective MR blockade and even distribution between the heart and kidneys help protect both organs and address the causes of cardiorenal syndromes often found in HFpEF patients [1]. Finerenone also has a lower risk of hyperkalemia and endocrine side effects than steroidal MRAs, which can make it easier for patients to tolerate, safer for long-term use, and may help with adherence[5].

Given the mechanistic rationale and pharmacokinetic advantages, finerenone emerges as an attractive option for chronic HFpEF management, particularly among patients with diabetic kidney disease. The potential for long-term disease modification supports its incorporation into evolving heart failure treatment paradigms[7].

## 4 MECHANISTIC INSIGHTS INTO FINERENONE’S ACTIONS IN HFPEF

### 4.1 Effects on Cardiac Remodeling and Fibrosis

Finerenone has a strong impact on cardiac remodeling in HFpEF, mainly by reducing myocardial fibrosis and hypertrophy. Clinical and pre-clinical studies show that finerenone lowers collagen buildup, reduces fibrotic signaling, and lessens harmful cardiac hypertrophy, which helps improve how the heart muscle works [3]. In mouse models of HFpEF caused by obesity and high blood pressure, finerenone treatment greatly reduces the size of heart muscle cells and improves mitochondrial protein acetylation, a sign of better mitochondrial function, without changing blood pressure. This suggests that its heart-protective effects do not depend on lowering blood pressure[4, 7].

These antifibrotic and anti-inflammatory actions correspond with reduced oxidative stress and inhibition of pro-inflammatory mediators within cardiac tissues, contributing to preserved cardiac structure and function in HFpEF[8]. This evidence underlines the potential of finerenone to address the molecular and cellular underpinnings of HFpEF beyond symptomatic relief.

### 4.2 Influence on Natriuretic Peptides and Hemodynamics

N-terminal pro-B-type natriuretic peptide (NT-proBNP) is an important biomarker in heart failure because it shows how much stress the heart muscle is under and helps predict patient outcomes. The FINEARTS-HF trial found that finerenone treatment lowers NT-proBNP levels by about 12% within a few months compared to placebo in patients with HFmrEF and HFpEF, regardless of their starting NT-proBNP levels [10]. This change in the biomarker is linked to better clinical results, such as fewer deaths from cardiovascular causes and fewer heart failure events.

Preclinical and clinical studies show that finerenone improves cardiac hemodynamics and vascular remodeling. In animal models of HFpEF, it reduces aortic medial thickness and vascular fibrosis, which points to better arterial compliance and less vascular stiffness[11]. Echocardiographic studies also confirm that finerenone has a positive effect on heart structure, with lower left atrial volume index and better diastolic function, such as improved E/e'' ratios, in patients who receive finerenone[6]. Together, these results suggest that finerenone helps prevent harmful changes in the heart and blood vessels, leading to better hemodynamic performance.

### 4.3 Interaction with Renal Function and Electrolytes

Finerenone can cause a small initial drop in estimated glomerular filtration rate (eGFR), similar to what happens with renin-angiotensin-aldosterone system inhibitors. It is important to consider this change in the clinical context so treatment is not stopped too early. In the FINEARTS-HF study, a 15% decrease in eGFR after one month was linked to a higher risk of negative outcomes, but it did not reduce how well finerenone worked. This finding supports continuing treatment with careful monitoring[12].

Managing serum potassium is important because MR antagonists can increase the risk of hyperkalemia. When patients were grouped by their starting potassium levels and kidney function, finerenone was equally effective at reducing heart failure events in all groups. Patients with lower kidney function or higher potassium had more side effects and stopped treatment more often, but these issues did not change the overall benefit of finerenone [13]. Finerenone also helped reduce fluid overload and heart enlargement through some mechanisms that do not depend on lowering blood pressure, showing its broad therapeutic effects[11].

Finerenone blocks the mineralocorticoid receptor at the molecular level, stopping the transcription of genes that cause inflammation and fibrosis. This action reduces macrophage infiltration, fibroblast activation, and buildup of the extracellular matrix. As a result, the heart becomes more flexible, the ventricles are less stiff, and damage to the heart and kidneys is lessened. Finerenone is different from other mineralocorticoid receptor antagonists because it has anti-inflammatory, antifibrotic, and kidney-protective effects. The graphical abstract compares finerenone to traditional steroidal mineralocorticoid receptor antagonists, showing differences in receptor selectivity, tissue distribution, effects on inflammation and fibrosis, and safety.

## 5 CLINICAL TRIAL EVIDENCE OF FINERENONE IN HFPEF

### 5.1 Overview of Key Clinical Trials: FIDELIO-DKD, FIGARO-DKD, and FINEARTS-HF

Finerenone has been studied in several major randomized controlled trials. The FIDELIO-DKD and FIGARO-DKD trials mainly included patients with diabetic kidney disease and showed that finerenone significantly reduced cardiovascular and kidney problems, including hospitalizations for heart failure [1]. These studies also found benefits for patients with preserved ejection fraction who had both chronic kidney disease and diabetes, suggesting that finerenone could be useful for a wider group of patients.

FINEARTS-HF is a large, international phase III trial that tested how well finerenone works and how safe it is for people aged 40 and older who have heart failure with mildly reduced or preserved ejection fraction (LVEF 40%)[14, 15]. To join the study, patients needed to have high natriuretic peptide levels and signs of structural heart disease. The main outcome measured was a combination of all worsening heart failure events (both first and repeat) and deaths from cardiovascular causes. The trial included 6001 patients from around the world, allowing researchers to study both HFpEF and HFmrEF[15].

FIDELIO-DKD and FIGARO-DKD provide useful information about shared inflammatory and profibrotic mechanisms. However, we should not assume that results from diabetic chronic kidney disease groups apply directly to HFpEF patients. These trial findings are best viewed as a starting point for new ideas, not as final evidence for treating HFpEF.

### 5.2 Efficacy Outcomes in HFpEF and HFmrEF

In the FINEARTS-HF study, finerenone consistently lowered the risk of the main composite outcome in all LVEF groups: those with LVEF below 50%, between 50% and 60%, and 60% or higher. The rate ratios ranged from 0.80 to 0.94, and there was no significant difference by ejection fraction, showing similar effectiveness across all subgroups [14, 15]. Most of the benefit came from fewer total worsening heart failure events. Prespecified analyses have confirmed these results, showing a steady reduction in cardiovascular death and hospitalizations, regardless of ejection fraction type[14, 15].

### 5.3 Safety and Tolerability in Clinical Trials

Safety analyses show that finerenone raises the risk of hyperkalemia and hypotension compared to placebo, mainly because it blocks mineralocorticoid receptors. However, the rates of treatment-emergent adverse events, serious adverse events, and discontinuations are similar [9]. Compared to eplerenone and spironolactone, finerenone causes fewer adverse events and less hyperkalemia, which supports its better tolerability [9]. The long-term safety results from FINEARTS-HF suggest that finerenone is suitable for ongoing therapy in HFpEF, as long as laboratory monitoring is in place.

## 6 REAL-WORLD EVIDENCE AND OBSERVATIONAL STUDIES

### 6.1 Finerenone Use in Diabetic Kidney Disease Patients with HFpEF

Studies in real-world settings show that finerenone is safe and helps support heart health in patients with diabetic kidney disease (DKD) and either HFpEF or HFmrEF. In a small group followed for six months, most people tolerated finerenone well. There was a small drop in eGFR and a slight rise in serum potassium, but these changes evened out, and hospitalizations for hyperkalemia were rare [6]. Echocardiograms also showed some improvements, like a lower left atrial volume index and a reduced E/e’’ ratio, which suggest better heart function and structure.

Moreover, most patients concurrently received sodium-glucose cotransporter 2 inhibitors (SGLT2i), with no adverse interaction detected, suggesting the feasibility of combination therapy in routine practice [16].

### 6.2 Demographic and Clinical Characteristics Affecting Outcomes

When looking at different age groups, finerenone shows similar clinical benefits and safety for both older and younger people with HFpEF, making it suitable for people with this condition [14, 15]. The FINEARTS-HF trial also found that women and men respond similarly to finerenone, even though they may have different heart and kidney characteristics at the start [14, 15]. In addition, having other health problems like chronic obstructive pulmonary disease (COPD), which usually makes HFpEF worse, does not reduce the benefits of finerenone. This suggests that finerenone could help a wide range of patients with different clinical backgrounds [14, 15].

### 6.3 Limitations and Knowledge Gaps in Observational Data

Real-world data often have small sample sizes, short follow-up periods, and potential selection biases, which limit the extent to which the results can be generalized [6]. Larger and more diverse prospective studies are needed to confirm these results and better understand long-term safety and effectiveness, especially in people with HFpEF who do not have diabetes or CKD [14, 15]. It is important to interpret these findings carefully to avoid overstating the benefits or missing rare side effects that can occur in observational studies.

## 7 COMPARATIVE ANALYSIS OF FINERENONE AND STEROIDAL MRAS

### 7.1 Efficacy Differences Across Heart Failure Types

Steroidal MRAs such as spironolactone clearly reduce deaths and hospital stays, especially in patients with heart failure with reduced ejection fraction (HFrEF), but the evidence is weaker for HFpEF[1]. Meta-analyses show these drugs work best in HFrEF and become less effective as LVEF rises. Finerenone, however, appears to benefit patients with HFpEF and HFmrEF too, offering more treatment choices [9]. Studies comparing the two drugs suggest finerenone is at least as effective as spironolactone for important outcomes, although there are no direct randomized trials between them[9].

### 7.2 Safety Profiles and Side Effect Burden

Steroidal MRAs are limited by hyperkalemia, renal dysfunction, and off-target endocrine effects. In contrast, finerenone is associated with a significantly lower incidence of treatment-emergent and serious adverse events, including hyperkalemia, as demonstrated in clinical trial data comparing it to eplerenone and spironolactone[9]. Furthermore, finerenone has less pronounced effects on blood pressure, reducing the risk of hypotension and related complications[7].

### 7.3 Clinical Guidance for Therapeutic Choice

When choosing an MRA, clinicians consider factors like how well patients tolerate steroid side effects, kidney function, potassium levels, and other health conditions. Finerenone is becoming a good option for patients who cannot take steroidal MRAs, especially those with CKD or diabetes [1]. Its use in guideline-based heart failure treatment, particularly for HFpEF, is increasing as more research and real-world data support its benefits [17]. Ongoing combination trials with other drugs aim to improve patient-focused treatment plans[16].

## 8 COMBINATION THERAPY WITH FINERENONE AND OTHER HEART FAILURE TREATMENTS

### 8.1 Use with Sodium-glucose Cotransporter 2 Inhibitors (SGLT2i)

SGLT2 inhibitors and finerenone have both been shown to reduce cardiovascular and kidney events in patients with HFpEF and T2DM. Subgroup analyses from FINEARTS-HF show that finerenone’s benefits occur regardless of whether patients are already using SGLT2 inhibitors, and that using both drugs together may offer additional protection against cardiovascular events [16]. SGLT2 inhibitor therapy may also lower the risk of hyperkalemia associated with finerenone, potentially making combined treatment safer. These results support using both drugs together to take advantage of their different ways of working.

### 8.2 Potential Role with Angiotensin Receptor Neprilysin Inhibitors (ARNI)

Using both MRAs and ARNIs together is a logical way to improve treatment results in heart failure. Early research suggests that adding finerenone to ARNIs may offer additional benefits, as they target distinct molecular pathways and have combined effects on heart structure and blood flow [18]. Current studies are examining the optimal order for these drugs, which patients are most suitable for, and how well people tolerate them, to help guide their use in practice [14, 15]. It is important to monitor kidney function and electrolyte levels, especially in patients at higher risk.

### 8.3 Emerging Combinations and Future Directions

Researchers are now studying combinations that include glucagon-like peptide-1 receptor agonists (GLP-1RAs) with SGLT2 inhibitors and finerenone. These combinations aim to use their multiple metabolic and anti-inflammatory effects to better protect the heart and kidneys [14, 15]. New selective MR modulators and therapies that work in several ways also show promise for treating heart failure. Current research focuses on finding the best combinations, timing, and patient types to get the most clinical benefit[16].

### 8.4 Safety Considerations and Management of Adverse Effects

Hyperkalemia is the most common and serious side effect of mineralocorticoid receptor antagonists (MRAs). Finerenone increases this risk compared to placebo, so it is important to monitor serum potassium levels regularly, especially in patients with kidney disease or those taking other medicines that increase potassium levels. To lower the risk, adjust the dose, offer dietary advice, or use potassium-lowering treatments as needed. Carefully selecting patients and checking labs frequently can make treatment safer and more effective. Regularly checking kidney function also helps prevent complications.

 Finerenone can cause low blood pressure more often than a placebo, but this side effect is usually manageable and rarely leads patients to stop treatment [9]. Some patients may see a drop in eGFR at first, but it often levels out and does not harm long-term kidney health, so treatment can continue with a doctor’s supervision [12]. Adjusting the dose and monitoring for signs of low blood pressure helps keep treatment safe for patients who are at risk.

 Elderly patients and people with several health conditions tolerate finerenone about the same across different age groups, with no major rise in side effects or stopping treatment [14]. Studies by gender show that women and men have similar safety results, even though their bodies differ in some ways [14, 15]. Also, having lung diseases like COPD does not increase the risks linked to finerenone, which supports its use in a wide range of real-world patients.

## 9 DISCUSSION

Heart failure with preserved ejection fraction (HFpEF) is a complex condition that is difficult to treat, with a wide range of causes and symptoms. Finerenone is a promising treatment option, but its effects should be interpreted carefully and not just based on trial results.

Randomized trials show that finerenone consistently lowers the risk of worsening heart failure, but its effect on cardiovascular death is smaller. This may be because the trials were short, people with HFpEF often die from other causes, and the disease itself is complex. These findings suggest that finerenone primarily slows disease progression and reduces inflammation and fibrosis in the heart, rather than providing a quick survival benefit.

HFpEF is now seen as a syndrome made up of different biological phenotypes, not just one disease. Patients with inflammatory-fibrotic or cardiorenal-dominant types may benefit more from mineralocorticoid receptor antagonists than those with mainly volume-overload or atrial-functional disease. Finerenone has shown consistent effectiveness across ejection fraction groups in FINEARTS-HF, suggesting it can be widely used. Still, we do not fully understand how different biological phenotypes affect treatment response, so more research is needed.

Most early data on finerenone comes from the FIDELIO-DKD and FIGARO-DKD trials, which mainly looked at kidney outcomes in people with type 2 diabetes and chronic kidney disease. These studies are important because they highlight shared pathways such as mineralocorticoid receptor overactivation, inflammation, and fibrosis. Still, it is important to be cautious when applying these findings to HFpEF. The FINEARTS-HF trial, on the other hand, is the first large randomized study to test finerenone in people with symptomatic HFpEF and HFmrEF, giving direct evidence of its benefit for this group.

The current evidence has some key limitations. Most clinical benefits rely on composite endpoints, and the long-term effects on mortality are still unknown. There are also no direct comparisons with steroidal mineralocorticoid receptor antagonists. Future studies should track patients over longer periods, look more closely at different subgroups, and find better ways to combine treatments with agents such as sodium-glucose cotransporter-2 inhibitors. These steps could help show which patients benefit most and lead to better treatment results.

Overall, finerenone emerges as a rational, well-tolerated therapy that addresses key biological mechanisms underlying HFpEF, with growing evidence supporting its integration into contemporary heart failure management.

## 10 FUTURE RESEARCH DIRECTIONS AND CLINICAL IMPLICATIONS

 Current studies are looking at how finerenone can be used for more than just diabetic kidney disease. Researchers are testing its effects in people with non-diabetic HFpEF and other cardiorenal conditions. They are focusing on long-term results like mortality, hospital stays, and kidney outcomes to better understand where finerenone fits as a treatment. These studies will also give information about using finerenone with other therapies and its safety in a wider range of patients.

Finding biomarkers and imaging features that predict how patients respond to finerenone is important for choosing the right patients. Adjusting finerenone treatment to fit the different types of HFpEF can improve results and reduce side effects. Using precision medicine, such as genetic and molecular profiling, will likely help create more personalized treatment plans and improve patient outcomes.

 Finerenone is now included in recent clinical guidelines for treating diabetic chronic kidney disease and heart failure with preserved or mildly reduced ejection fraction. Efforts are ongoing to promote its wider use in clinical practice, depending on growing evidence and clinician education. Cost-effectiveness, access, and the impact on healthcare systems are still being studied to help ensure fair access to this therapy.

## CONCLUSION

 FINEARTS-HF represents the first large-scale randomized trial specifically designed to evaluate finerenone in patients with heart failure with mildly reduced or preserved ejection fraction, establishing direct outcome evidence beyond extrapolation from diabetic kidney disease populations.

Finerenone is effective for people with different levels of heart function, even if their heart function is only slightly reduced or still normal. It is safer than steroidal mineralocorticoid receptor antagonists (MRAs) because it leads to less hyperkalemia and fewer hormone-related side effects. These benefits are especially important for older adults, women, and people with lung problems.

Finerenone is becoming an important new therapy for managing HFpEF, helping to meet a long-standing clinical need. When used with established treatments like SGLT2 inhibitors, it may offer added cardiorenal protection. This makes finerenone a valuable part of strategies to improve outcomes for high-risk patients with complex health issues.

 Continuing research helps us choose the right patients, find the best treatment combinations, and learn more about the long-term effects of finerenone. Regular checks for high potassium and kidney function are needed to get the most benefit and reduce risks. As finerenone is used more often for cardiorenal disease, it will likely play a bigger role in patient care.

## AUTHORS' CONTRIBUTIONS

The authors confirm their contribution to the paper as follows: S.G.S. conceptualized the study, performed the literature review, synthesized trial data, drafted and critically revised the manuscript. D.M. provided senior supervision, expert input on manuscript design, and critical revision for intellectual content. Both authors approved the final version for submission.

## LIST OF ABBREVIATIONS

ARNI: Angiotensin Receptor-Neprilysin Inhibitor
CKD: Chronic Kidney Disease
eGFR: Estimated Glomerular Filtration Rate
GLP-1RA: Glucagon-Like Peptide-1 Receptor Agonist
HFpEF: Heart Failure with Preserved Ejection Fraction
HFrEF: Heart Failure with Reduced Ejection Fraction
MRA: Mineralocorticoid Receptor Antagonist
NT-pro BNP: N-terminal pro-B-type Natriuretic Peptide
SGLT2i: Sodium-Glucose Cotransporter-2 Inhibitor
T2DM: Type 2 Diabetes Mellitus
